# Supplementary material for: Progressive degeneration in a new Drosophila model of spinocerebellar ataxia type 7
Source: Sci Rep. 2024 Jun 21;14:14332. doi: 10.1038/s41598-024-65172-4 (PMC11192756; doi:10.1038/s41598-024-65172-4)
Supplement: Supplementary file 1 — Supplementary Information. [file 41598_2024_65172_MOESM1_ESM.pdf]

Supplemental figure 1

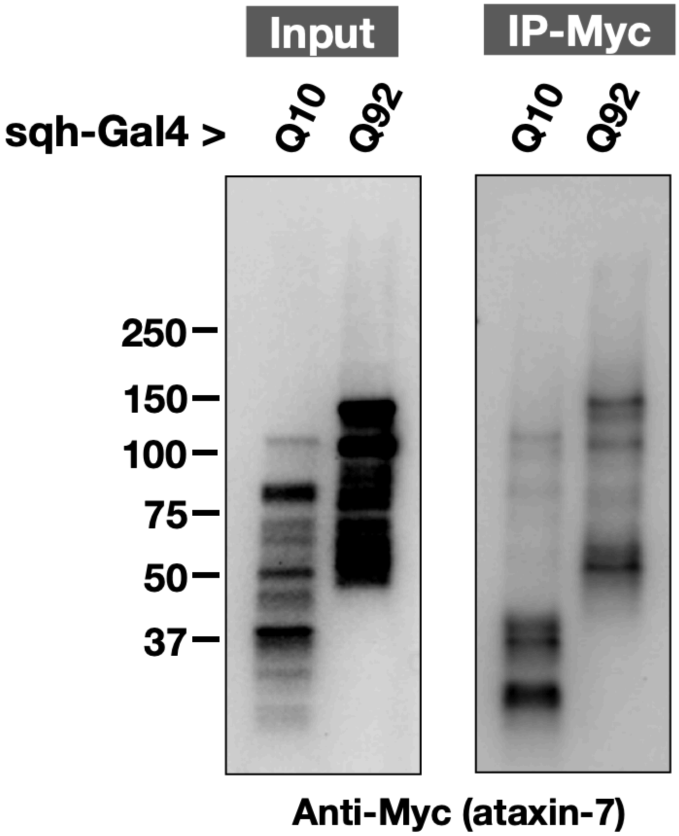

Immunoprecipitation of Myc-tagged ATXN7.
Shown are Western blots of flies expressing ATXN7 with Q10 or Q92 in all tissues. Inputs are on the left and IPs are on the right. Blots were probed with anti ATXN7 antibody.

Supplemental figure 2

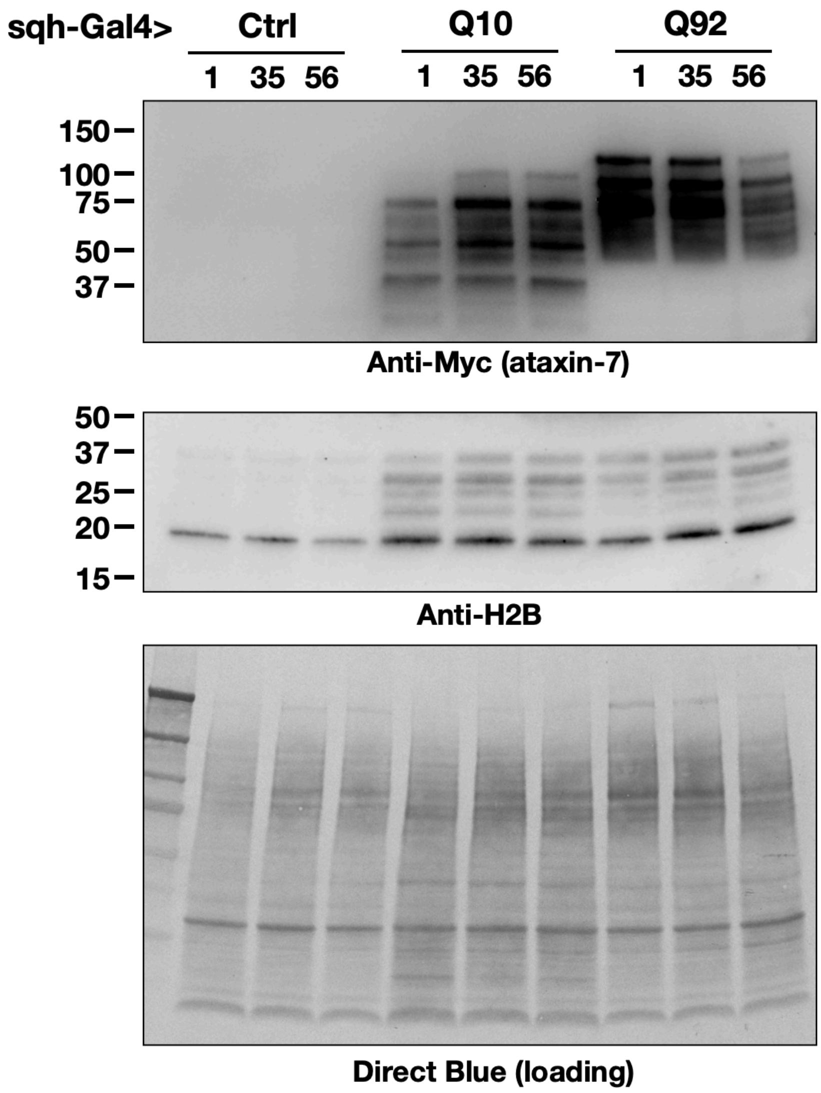

Levels of H2B when ATXN7 is expressed in all tissues.
Shown are Western blots from whole fly lysates where ATXN7 with Q10 or Q92 was expressed via sqh-Gal4. Flies were one day old. Ctrl had the driver on the background used to generate SCA7 flies.

Supplemental figure 3

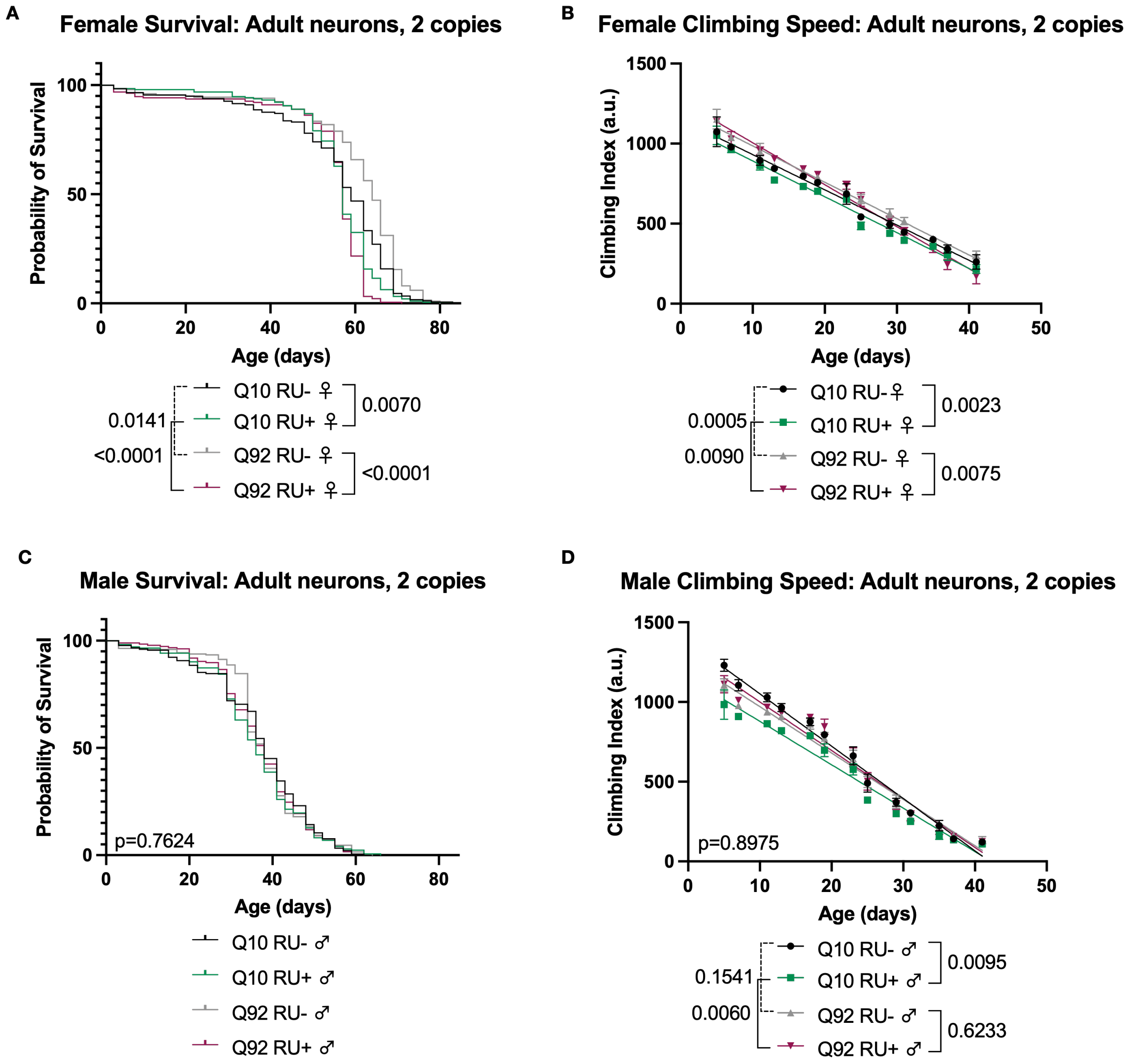

Longevity and motility outcomes from flies containing one or two copies of UAS-ATXN7, driven in all adult neurons.

The driver used was elav-GAL4-GS (GeneSwitch), allowing for transgene expression only in the presence of the inducer, RU486 (RU+). Flies were reared in food without RU486 (RU-) until the day they eclosed from the pupal case, then switched to media with RU486 until they died. Longevities and motilities were calculated as explained in the main article.

Supplemental figure 4

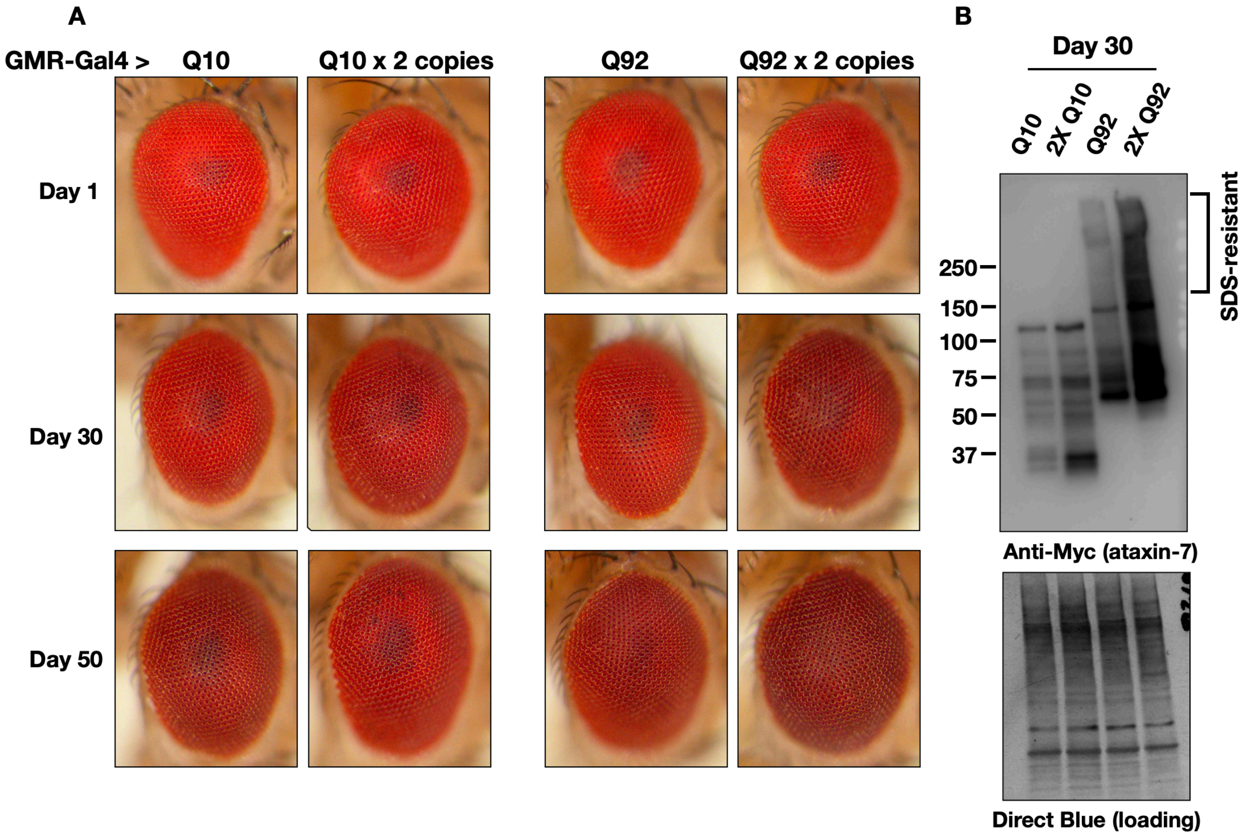

Effects of expressing one or two copies of UAS-ATXN7 in fly eyes.
A) representative photos of external eyes with the noted genotypes. B) Western blots from flies with the genotypes as in (A).

Supplemental figure 5

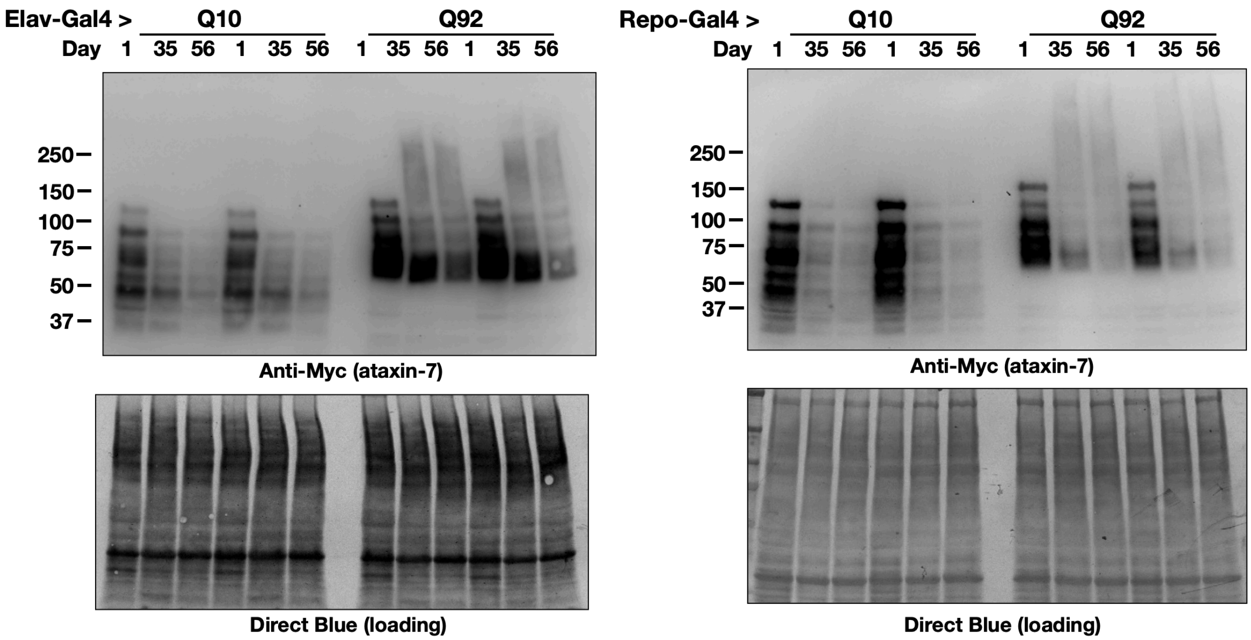

Migration of ATXN7 species in neuronal and glial tissues over time.
Western blots from flies expressing the noted transgenes in all neurons (left) or glia (right) over the course of time.

Supplemental figure 6

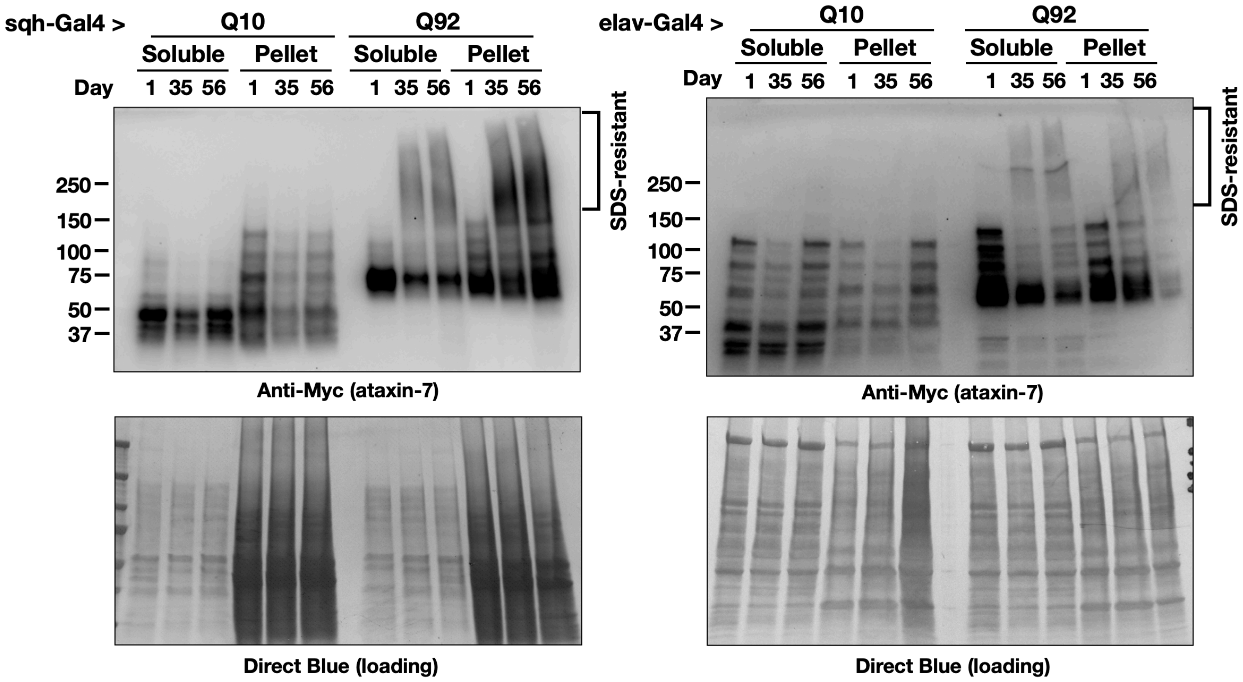

Partitioning of ATXN7 species in soluble and pellet fractions when expressed in all neuronal cells.

Western blots from flies expressing Q10 or Q92 ATXN7 in all neuronal cells, processed as described in the methods for soluble and pellet fractions.

Supplemental figure 7

Full-length gels and un-cropped blots in figures

Supplemental figure 7 (un-cropped Figure 1B)

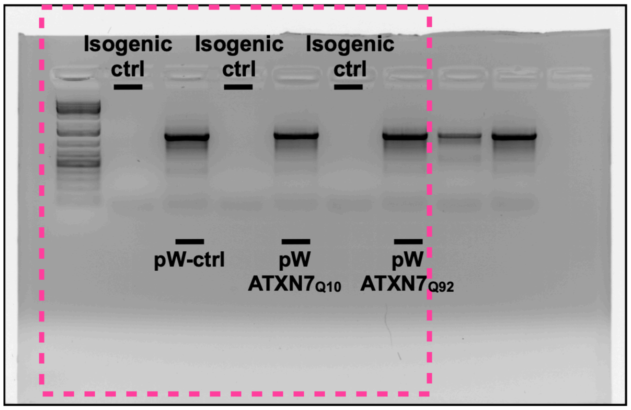

Supplemental figure 7 (un-cropped Figure 1C)

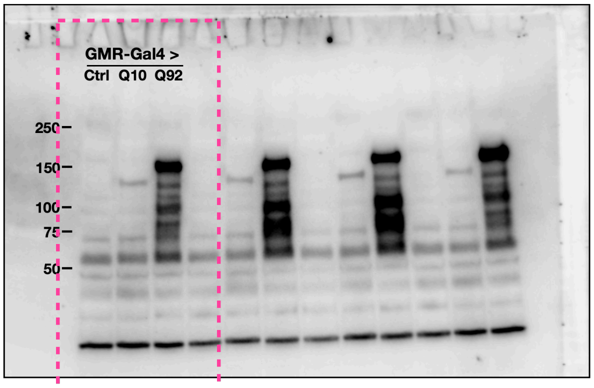

Anti-Myc (ataxin-7)

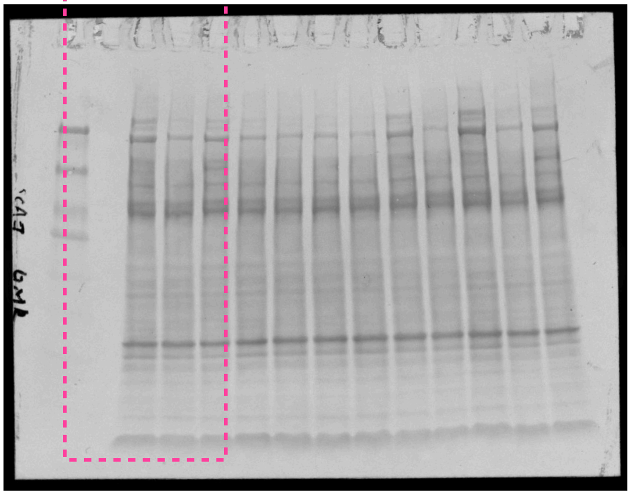

Direct Blue (loading)

Supplemental figure 7 <u>(un-cropped Figure 1D)</u>

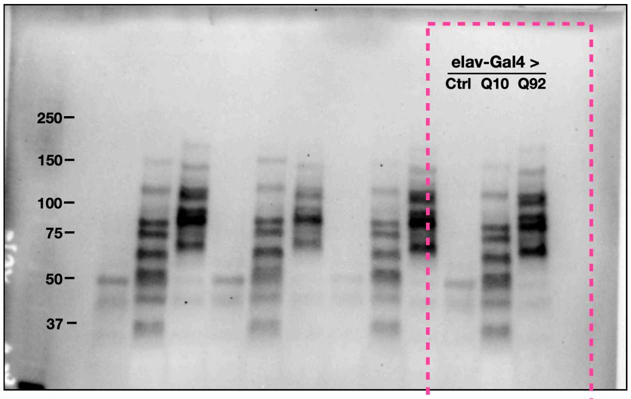

Anti-Myc (ataxin-7)

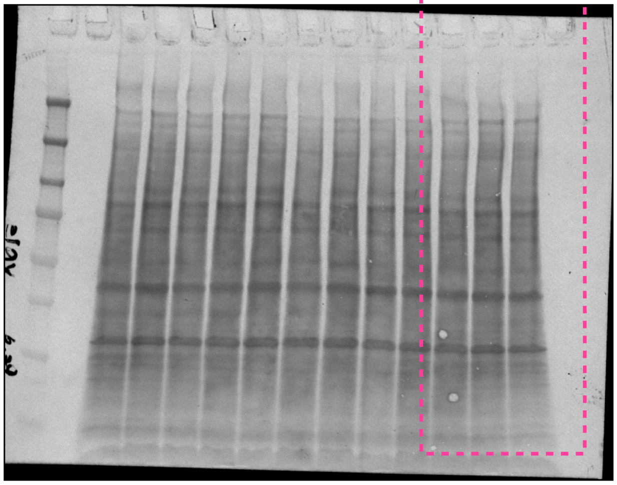

Direct Blue
(loading)

Supplemental figure 7
<u>(un-cropped Figure 1E)</u>

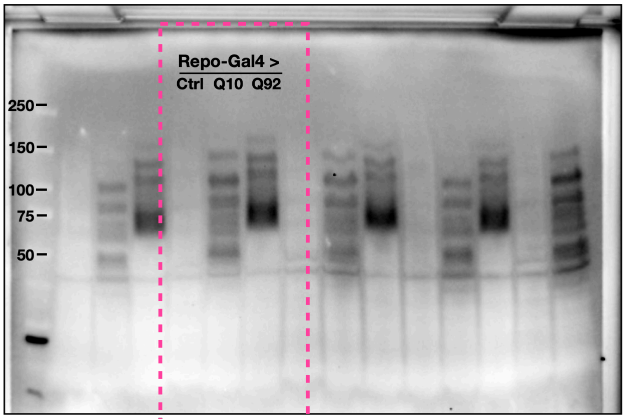

Anti-Myc (ataxin-7)

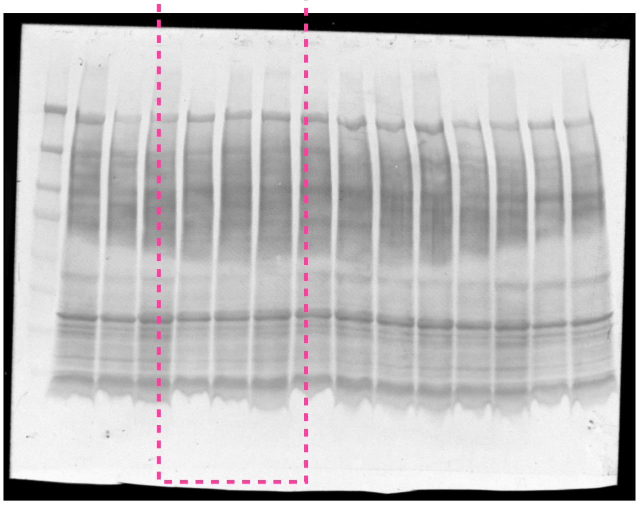

Direct Blue
(loading)

Supplemental figure 7 (un-cropped Figure 1F)

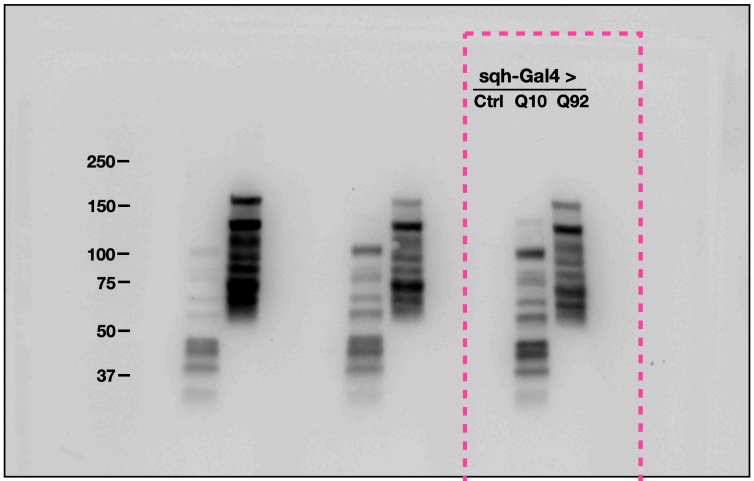

Anti-Myc (ataxin-7)

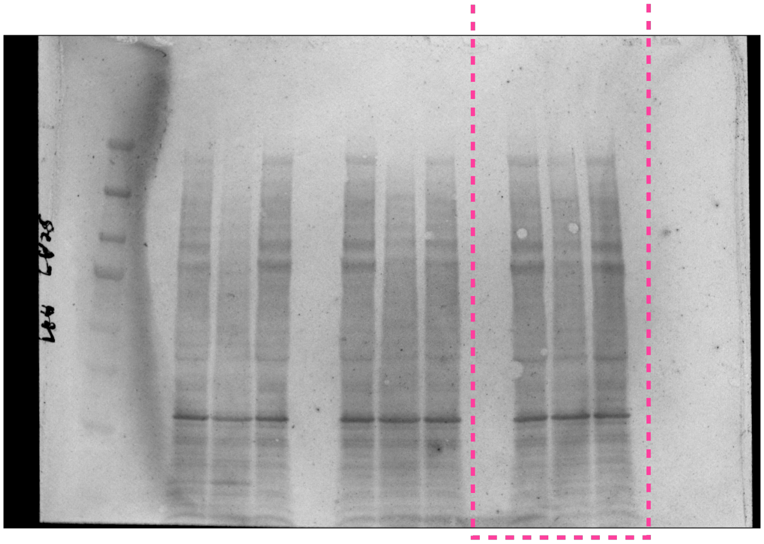

Direct Blue
(loading)

Supplemental figure 7 (un-cropped Figure 4C)

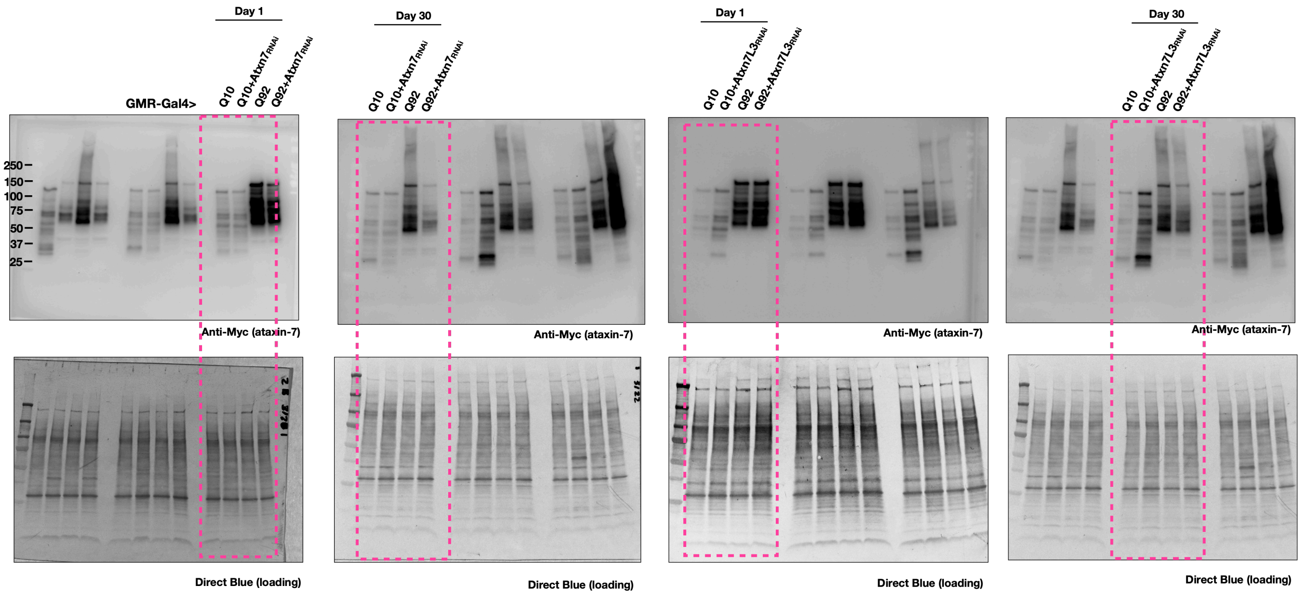

Supplemental figure 7 (un-cropped Figure 5C)

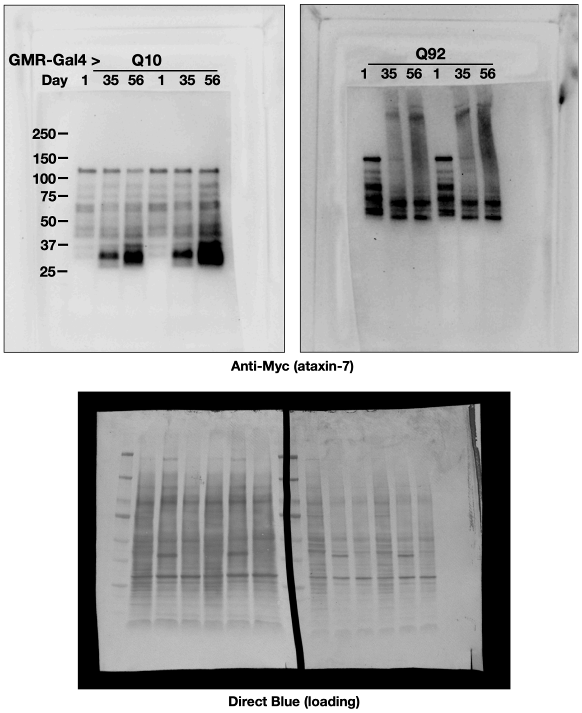

Supplemental figure 7 (un-cropped Figure 5D)

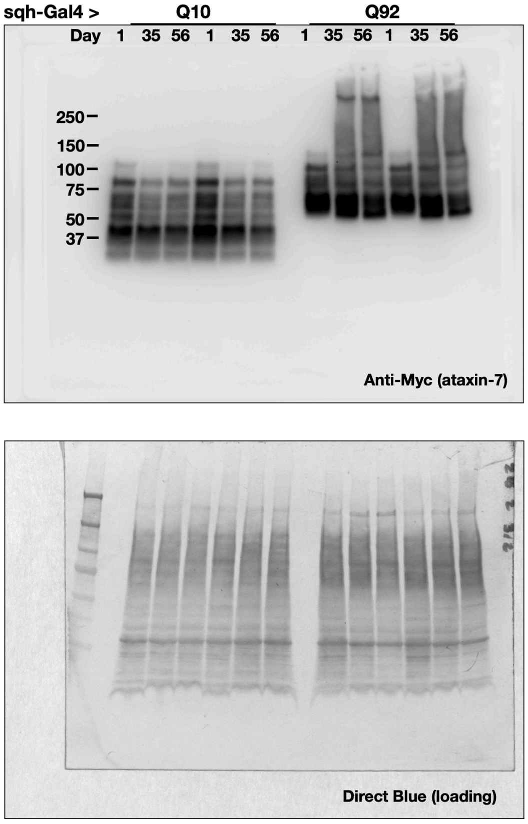

Supplemental figure 7 (un-cropped Supplemental figure 1)

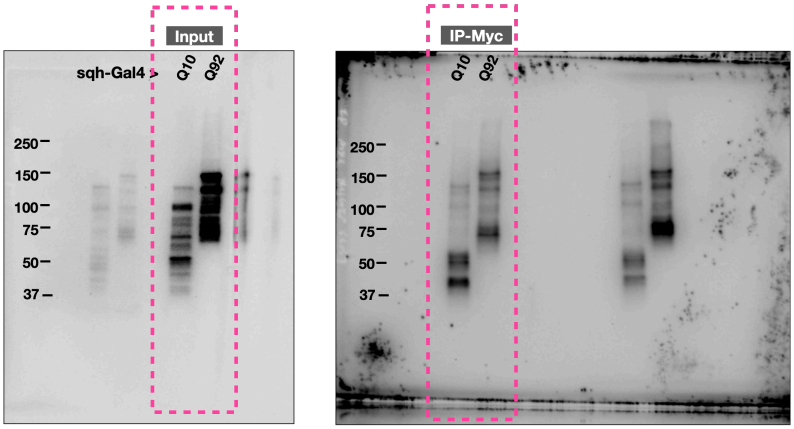

Anti-Myc (ataxin-7)

Anti-Myc (ataxin-7)

Supplemental figure 7 (un-cropped Supplemental figure 2)

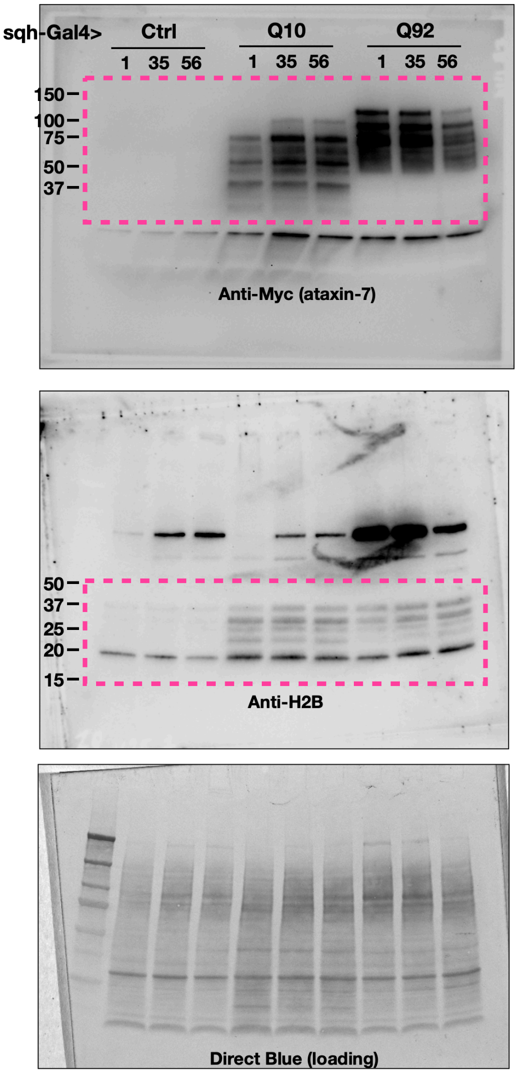

Supplemental figure 7 **<u>(un-cropped Supplemental figure 4B)</u>**

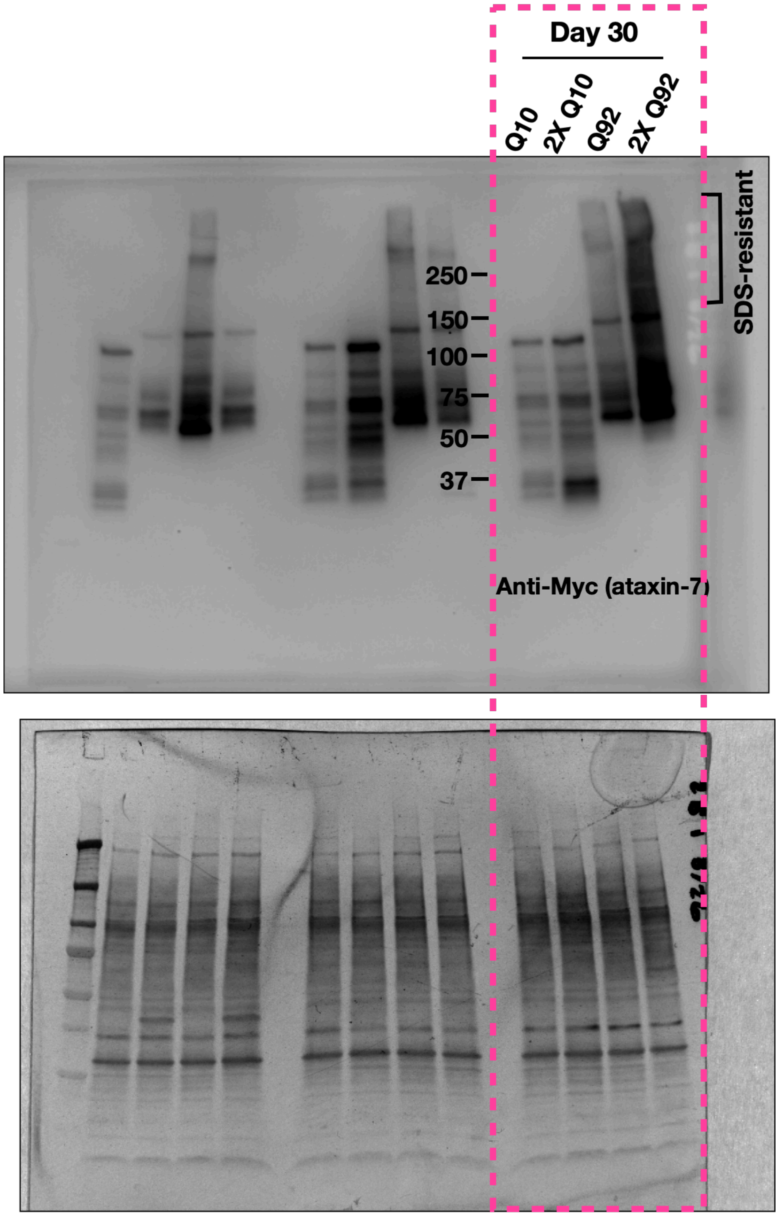

Direct Blue (loading)

Supplemental figure 7 (un-cropped Supplemental figure 5)

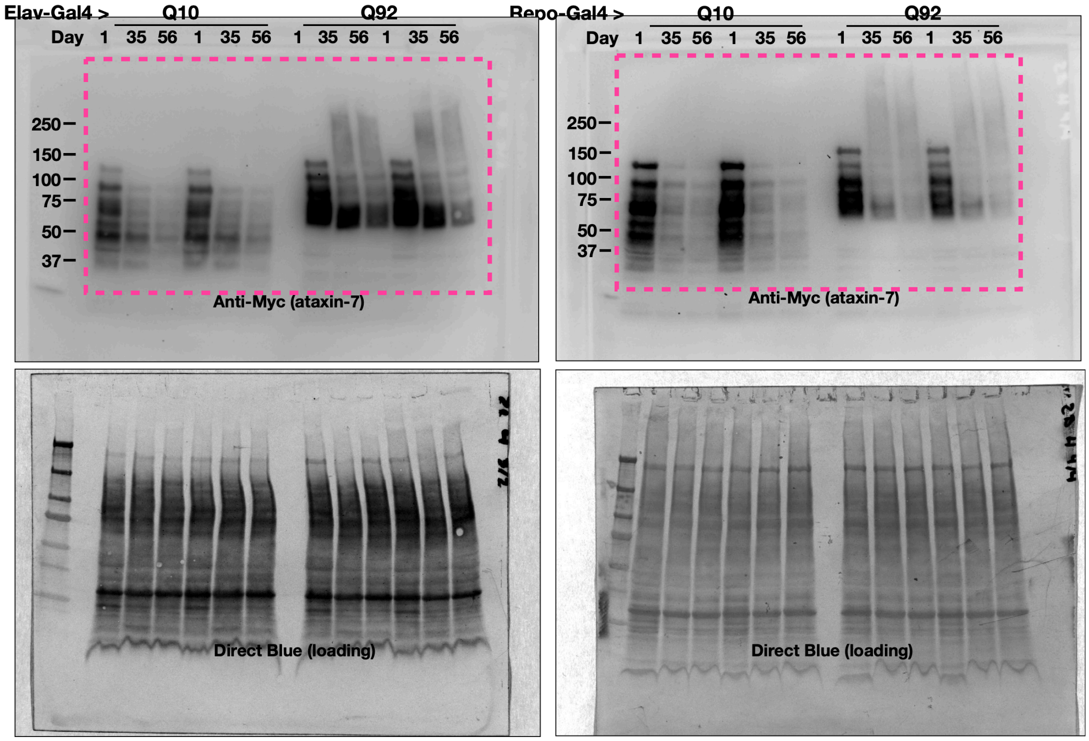

Supplemental figure 7 (un-cropped Supplemental figure 6)

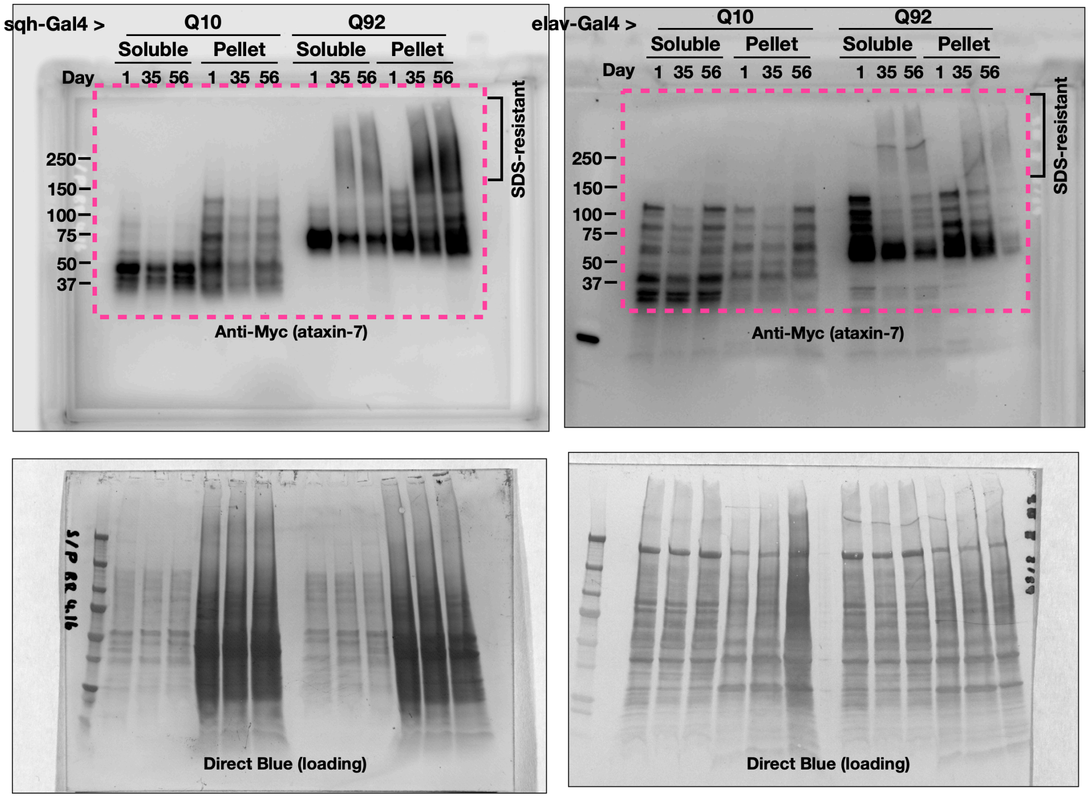

Supplemental Table 1

List of antibodies used for Western blots

Name	Manufacturer	Catalog number	Concentrations used
**Myc-Tag (9B11)**	Cell Signaling	2276	1:1000
**Anti-Histone H2B**	Sigma-Aldrich	07-371	1:1000
**HRP-conjugated goat anti-mouse**	Jackson ImmunoResearch	115-035- 062	1:5000
**HRP-conjugated goat anti-rabbit**	Jackson ImmunoResearch	115-035- 045	1:5000
